# Isolation and antimicrobial resistance patterns of *Staphylococcus aureus* and *Escherichia coli* from caprine respiratory tract infections: A hospital-based clinical study

**DOI:** 10.5455/javar.2024.k855

**Published:** 2024-12-29

**Authors:** Shanta Barua, Md. Abu Sayeed, Md. Ashiqur Rahman, Mohammad Mahmudul Hassan, Mohammed Yousuf Elahi Chowdhury, Eaftekhar Ahmed Rana

**Affiliations:** 1 Department of Medicine and Surgery, Faculty of Veterinary Medicine, Chattogram Veterinary and Animal Sciences University, Chattogram, Bangladesh; 2 National Centre for Epidemiology and Population Health, College of Health and Medicine, The Australian National University, Canberra, Australia; 3 School of Veterinary Medicine, Murdoch University, Murdoch, Australia; 4 Department of Physiology, Biochemistry and Pharmacology, Faculty of Veterinary Medicine, Chattogram Veterinary and Animal Sciences University, Chattogram, Bangladesh; 5 Department of Microbiology and Veterinary Public Health, Faculty of Veterinary Medicine, Chattogram Veterinary and Animal Sciences University, Chattogram, Bangladesh

**Keywords:** Goats, *E. coli*, MDR, RTI, *S. aureus*

## Abstract

**Objective:**

*Staphylococcus aureus* and *Escherichia coli* are the most common opportunistic pathogens frequently associated with respiratory tract infection (RTI) in different animals. This cross-sectional study aimed to identify the occurrence of *S. aureus* and *E. coli* in goats with RTI, analyze the antimicrobial resistance patterns, and explore potential risk factors contributing to RTI.

**Materials and Methods:**

A total of 120 nasal swab samples were collected from goats, and standard classical bacteriological methods were performed to isolate and identify *S. aureus* and *E. coli*. Subsequently, the disc diffusion method was employed to evaluate the antimicrobial sensitivity test. A logistic regression model was used to analyze the factors associated with RTI.

**Results:**

About 13.3% (*N = *16; *N = *120) isolates were confirmed as *S. aureus,* and 6.67% (*N = *8; *N = *120) isolates were confirmed as *E. coli*. All S.* aureus* isolates were resistant to ampicillin, and all *E. coli* isolates were resistant to amoxicillin and penicillin. Among the isolated organisms, 43.7% (*N = *7; *N = *16) *S. aureus* and 62.5% (*N = *5; *N = *8) *E. coli *isolates were found to be multidrug-resistant (resistant to ≥3 classes of antimicrobials). Multivariable logistic regression analysis revealed that female goats [(odds ratio) OR: 4.2; 95% confidence intervals (CI): 0.8–20.8; *p* = 0.074] and goats in poor health condition (OR: 3.8; 95% CI: 0.7–19.3; *p* = 0.100) were more prone to RTI caused by *S. aureus*. Besides, goats that were not dewormed (OR: 4.8; 95% CI: 1–23.6; *p* = 0.051) and those reared in semi-intensive conditions (OR: 2.7; 95% CI: 0.8–8.7; *p* = 0.092) were found to be at higher risk of *S. aureus*-mediated RTI.

**Conclusion:**

The findings highlight the importance of implementing improved farm management practices and efficient antimicrobial resistance control approaches to minimize respiratory infections and reduce the burden of antibiotic resistance in goats.

## Introduction

Respiratory tract infection (RTI) is one of the most common illnesses of small ruminants, including goats, throughout the world. Multiple pathogens such as bacteria, viruses, and mycoplasma are the most common etiological agents associated with the respiratory disease complex in goats. Notably, different bacterial species such as *Streptococcus pneumoniae*, *Klebsiella pneumoniae*, *Pasteurella multocida*, *Mannheimia haemolytica, Acinetobacter baumannii*, *Escherichia coli*, *Staphylococcus aureus*, and *Pseudomonas aeruginosa* cause RTI [9,33]. Due to the high burden of opportunistic pathogens in both the environment and the host body, small ruminants like goats are more susceptible to infectious agents and often suffer from RTIs. However, RTI is manifested by different clinical symptoms such as high fever (104-106°F), mucopurulent nasal discharge, coughing, dyspnea, anorexia, and depression [45]. These clinical conditions are sometimes aggravated by various factors such as housing, climate, immune status, nutrition, associated co-infections, and so on [33]. Therefore, the clinical management of RTI goats creates a significant economic burden and hampers optimum production in goat farming. This disease condition is also considered one of the major constraints for goat farming in many developing countries, including Bangladesh [50]. Moreover, RTIs in goats are a major concern for global small ruminant production, impacting herd health and productivity by causing widespread illness and necessitating intensive veterinary care and management.

However, several antimicrobials, such as penicillin, ampicillin, amoxicillin, amoxicillin-clavulanic acid, third-generation cephalosporin, gentamicin, ciprofloxacin, and so on, are commonly used to treat RTIs in goats [15,28,9]. High antimicrobial selective pressure and irrational use mainly contribute to the evolution of antimicrobial resistance (AMR) among both obligatory and opportunistic bacterial pathogens [48]. Consequently, the significant threat to the clinical recovery of RTI goats that causes increased mortality rates hampers production and decreases profitability [36]. AMR is generally mediated by genetic mutation, resistance gene transfer, reduced target expression by bacteria, gene modification, alterations or modifications to drug binding sites, and the production of different enzymes against antimicrobials [48]. In selected areas of Bangladesh, *S. aureus* and *E. coli* isolated from large ruminants affected with RTI demonstrated resistance to several antimicrobials [4].

To date, limited studies have been conducted to determine the drug resistance pattern of respiratory pathogens in goats in some selected areas of Bangladesh. To the best of our knowledge, no study has been conducted in the Chattogram district to isolate and identify the opportunistic bacterial pathogens causing RTI in goats, identify potential risk factors of RTI, and explore their resistance patterns of commonly used antimicrobials. Therefore, the current study aimed to isolate and identify opportunistic respiratory pathogens, particularly *S. aureus* and *E. coli*, from RTI goats brought to SA Quaderi Teaching Veterinary Hospital (SAQTVH) in Chattogram, Bangladesh. In addition, the study determined the resistance patterns of commonly used antimicrobials and identified the potential risk factors associated with the occurrence of RTIs caused by opportunistic bacterial pathogens.

## Materials and Methods

### Ethical approval

The sampling procedure was non-invasive and conducted under the supervision of a registered veterinarian to minimize discomfort and stress to the goats. Before conducting the study and sample collection, verbal permission and consent from the SAQTVH director were taken. Informed consent was obtained from animal owners before nasal swab sample collection. All protocols were followed to ensure the welfare and safety of the animals throughout the study period, adhering to the ethical standards of both the teaching veterinary hospital and institutional animal research guidelines.

### Study design and period

**Figure 1.**
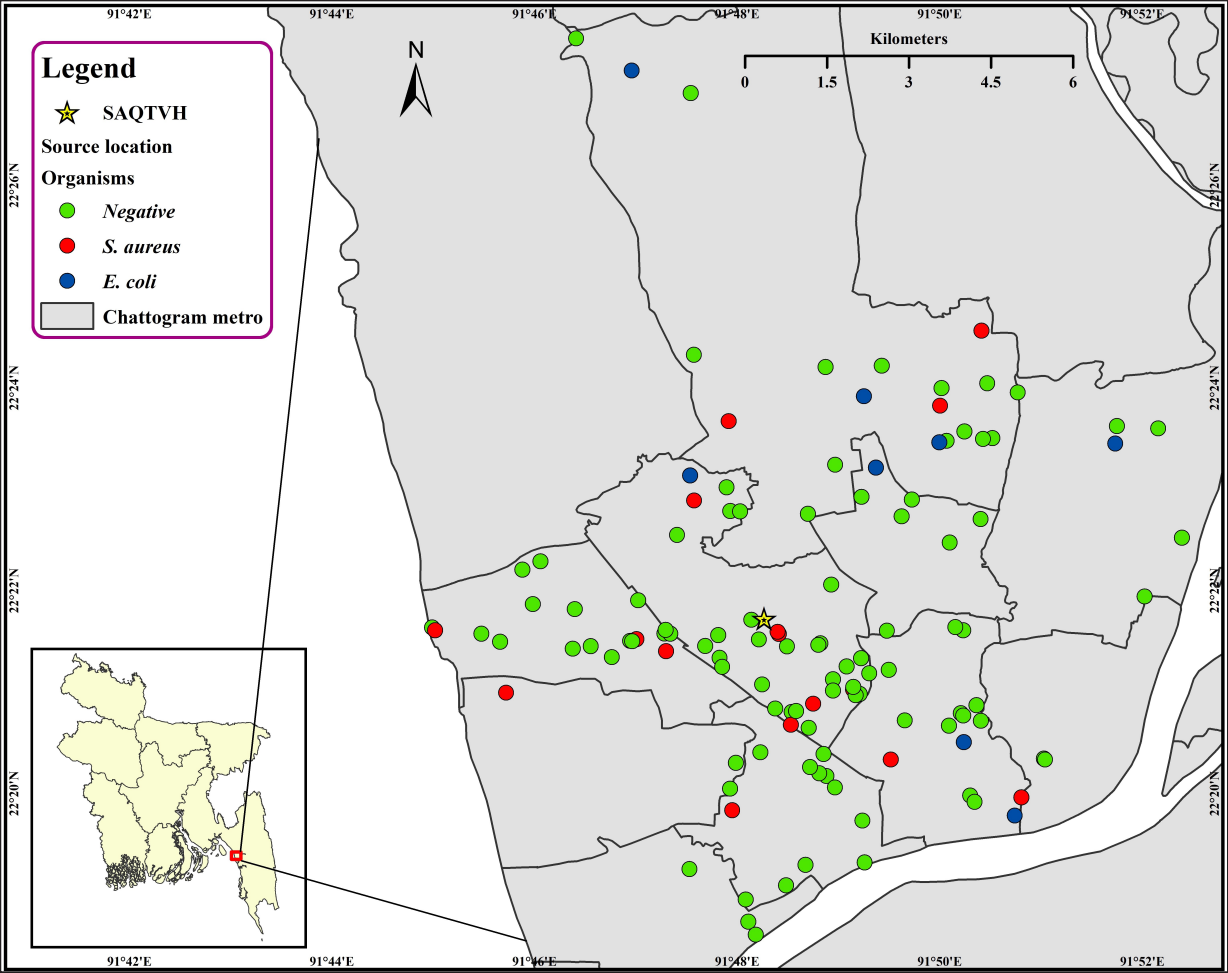
Geographical map represents the hotspot of RTI goats at Chattogram metropolitan region.

A cross-sectional study was conducted at SAQTVH, Chattogram Veterinary and Animal Sciences University (CVASU), from January to March 2021. All goats with RTIs were brought to SAQTVH from the Chattogram metropolitan area for diagnosis and treatment (Fig. 1).

### Animal selection criteria and sampling

During the study period, samples were collected only from goats exhibiting clinical respiratory symptoms, such as nasal discharge, coughing, sneezing, dyspnea, and respiratory distress, that had not been previously treated with antimicrobials. Only single samples were collected from each animal, with no repeat samples taken from the RTI-affected goats. In total, we collected 120 samples from RTI goats at the SAQTVH.

### Sample collection, preservation, and transportation

Samples were collected from goat nostrils using sterile cotton swab sticks (such as sterile cotton swab, model: PW005 by Hi Media Laboratories). The swab sticks were inoculated into 15 ml Falcon tubes containing 5 ml buffer peptone water (Oxoid, UK). After that, the samples were immediately transferred to the laboratory of the Department of Medicine and Surgery, CVASU, using an ice box to keep them at 4°C until further investigation.

### Data collection

A structured questionnaire was developed and administered to the goat owners to collect data related to the occurrence of RTI in goats. Parameters including the source of goats (farm or household), age (in months), sex (male or female), breed (local or Jamnapari), body condition score (BCS) (good or poor), rearing system (intensive or semi-intensive), vaccination (yes/no), deworming (yes/no), body temperature, and respiratory sound were considered for the study.

### Isolation, identification, and characterization of S. aureus

**Figure S1.**
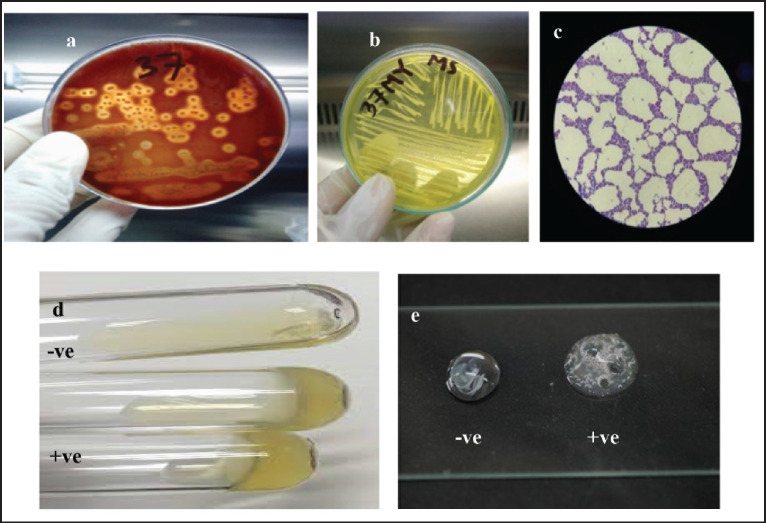
Confirmation of *S. aureus* on (a) β-hemolysis in blood agar plate, (b) Bright yellow colonies in mannitol salt agar plate, (c) Cluster of grapes like colonies of *S. aureus *on gram’s staining, (d) Ccoagulase test for *S. aureus, *and (e) Catalase test for *S. aureus.*

Nasal swab samples in buffer peptone water were incubated overnight at 37°C for enrichment. Bacterial enrichment was examined by the turbidity of the broth. Later, one loop of each cultured broth was streaked onto the blood agar plates using inoculating loops and incubated at 37°C for 24 h. On blood agar, *S. aureus* formed white to golden colonies with β-hemolysis (Supplementary Fig. 1a). The positive colonies were again streaked on selective media, namely, mannitol salt agar (MSA) plates*. *Then, the plates were incubated again overnight at 37°C. On MSA agar, *S. aureus* produced bright yellow colonies surrounded by yellow zones (Supplementary Fig. 1b). Later, the streak plate method was used to repeatedly subculture the colonies on primary cultures until homogenous, pure colonies were obtained. Finally, cultural characteristics and colony morphology of bacteria grown on blood and MSA agar were recorded.

Besides, Gram staining was performed to determine the cellular morphology and staining characteristics of the bacteria. In Gram’s staining, *S. aureus *positive isolates showed violet-colored cocci that were arranged in grape-like clusters (Supplementary Fig. 1c).

Additionally, isolates positive in culture media and Gram staining were subjected to biochemical tests, including catalase and coagulase tests, to confirm suspected *S. aureus* colonies (Supplementary Figs. 1d,e). A previously confirmed coagulase-positive *S. aureus *isolate was used as a positive control for the current study [41]. In the catalase and coagulase test, formation of effervescence and coagulation of horse plasma indicate *S. aureus* positive isolates.

### Isolation, identification, and characterization of E. coli

**Figure S2.**
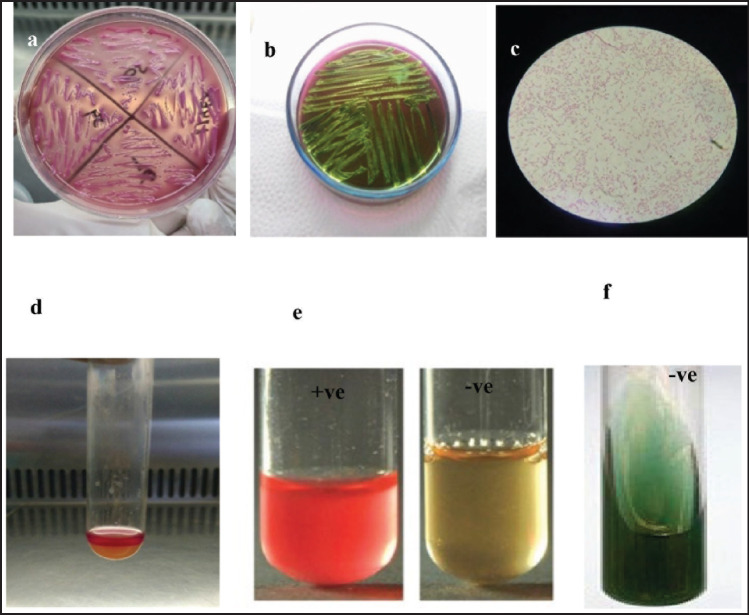
Confirmation of *E. coli* on (a) Pink colonies on MacConkey agar plate, (b) Metallic sheen on EMB agar plate, (c) Pink colored rod shaped colonies of *E. coli on *gram’s staining, (d) Indole test positive, (e) MR positive and VP negative, and (f) Simmons’ citrate negative.

On blood agar, *E. coli* formed large, off-white, and moist colonies. Then, the positive colonies were streaked on both MacConkey agar and eosin methylene blue (EMB) agar plates and incubated overnight at 37°C. On MacConkey agar, the bacteria formed rose-pink, lactose-fermented colonies. Besides, smooth, large, circular, blue-black colonies with a greenish metallic sheen were found on EMB agar (Supplementary Figs. 2a-b). Following that, the positive colonies were subcultured to obtain pure colonies. Later, Gram’s staining was performed. On Gram’s staining, *E. coli* produced a pink color, small, rod-shaped bacilli arranged in singles, pairs, or discreetly (Supplementary Fig. 2c). Finally, biochemical tests, namely the IMViC test [Indole, Methyl Red (MR), Voges Proskauer (VP), and Citrate test], were performed to confirm *E. coli* (Supplementary Figs. 2d,e,f). In the indole test, red-colored ring formation was noticed at the top of the tube after adding the Kovacs reagent [41]. In the MR test, the media color changed to red after adding the MR reagent, indicating positive *E. coli* isolates [41]. On the other hand, no color change after adding reagent in the VP media and lack of growth and color change of the citrate media indicates that the VP and citrate tests are negative, which further confirmed *E. coli *positive isolates.

### Antimicrobial resistance testing

Bacteriologically confirmed isolates were subjected to antimicrobial sensitivity testing. The standard disc diffusion procedure was used to carry out the screening of positive isolates against 11 different antimicrobial agents (Oxoid Ltd., Basingstoke, UK), such as CRO: Ceftriaxone (30 µg), CN: Gentamycin (10 µg), CIP: Ciprofloxacin (5 µg), ENR: Enrofloxacin (5 µg), TE: Tetracycline (30 µg), SXT: Sulfamethoxazole-trimethoprim (25 µg), AMC: Amoxicillin-Clavulanic acid (30 µg), AMP: Ampicillin (10 µg), AML: Amoxicillin (10 µg), AZM: Azithromycin (15 µg), and P: Penicillin (10 µg) from 6 different classes of drugs, including β-lactam, semi-synthetic β-lactam, tetracyclines, fluoroquinolones, sulfonamides, and aminoglycosides. Briefly, pure colonies of *S. aureus* and *E. coli* were suspended in sterile saline and standardized to the 0.5 McFarland standard separately. After that, pure bacterial suspension was uniformly spread on Muller-Hinton agar (Oxoid Ltd., Basingstoke, UK) plates with the help of sterile cotton swabs. Then, the selected antimicrobial disc was placed in the plates with the help of sterile forceps. Following that, the plates were incubated for 24 h at 37°C. After incubation, the plates were examined and the diameter of the zone of inhibition was measured. The result was interpreted as Resistant (R), Intermediate (I), and Sensitive (S) as per the guidelines of the Clinical and Laboratory Standard Institute [16]. Any isolate that showed resistance to three or more classes of antimicrobials was considered as multidrug-resistant (MDR) [7,47].

### Data analysis

Descriptive statistics were calculated to determine the frequency, percentage, and 95% confidence interval (CI) of both *S. aureus* and *E. coli* prevalence, AMR, and MDR patterns of isolated organisms. The univariate Fisher’s exact test was performed to determine the association between isolated bacteria (outcome) with different factors such as demographics (source, breed, age, and sex), health status (BCS), and management practices (rearing system, vaccination, and deworming) related to RTI in goats. Any variables having a *p*-value of ≤0.18 in Fisher’s exact test were selected for the subsequent multivariable logistic regression model. A forward stepwise selection approach was followed to calculate the final model. The collinearity among the factors was checked by the “Fisher’s Exact” test and the variations in the coefficients, along with the *p*-values. The model was then evaluated for goodness of fit using the Hosmer-Lemeshow test. For *E. coli*, no significant factors were identified in univariate analysis. Therefore, subsequent multivariate analysis was not performed. Finally, any associations having *a p-value* of ≤0.05 were considered significant for the final multivariate analysis of the model. All statistical analysis was performed using STATA^®^ 13.0 software.

## Results

### Occurrence of RTI caused by S. aureus and E. coli in goats

Among 120 swab samples, 13.3% (*N = *16) [95% CI: 7.8–20.7] isolates were confirmed as *S. aureus*, and 6.67% (*N = *8) (95% CI: 2.9–12.7) isolates were confirmed as *E. coli* based on cultural and biochemical characteristics.

### Clinical findings of RTI-infected goats (based on S. aureus and E. coli positive cases)

**Figure 2.**
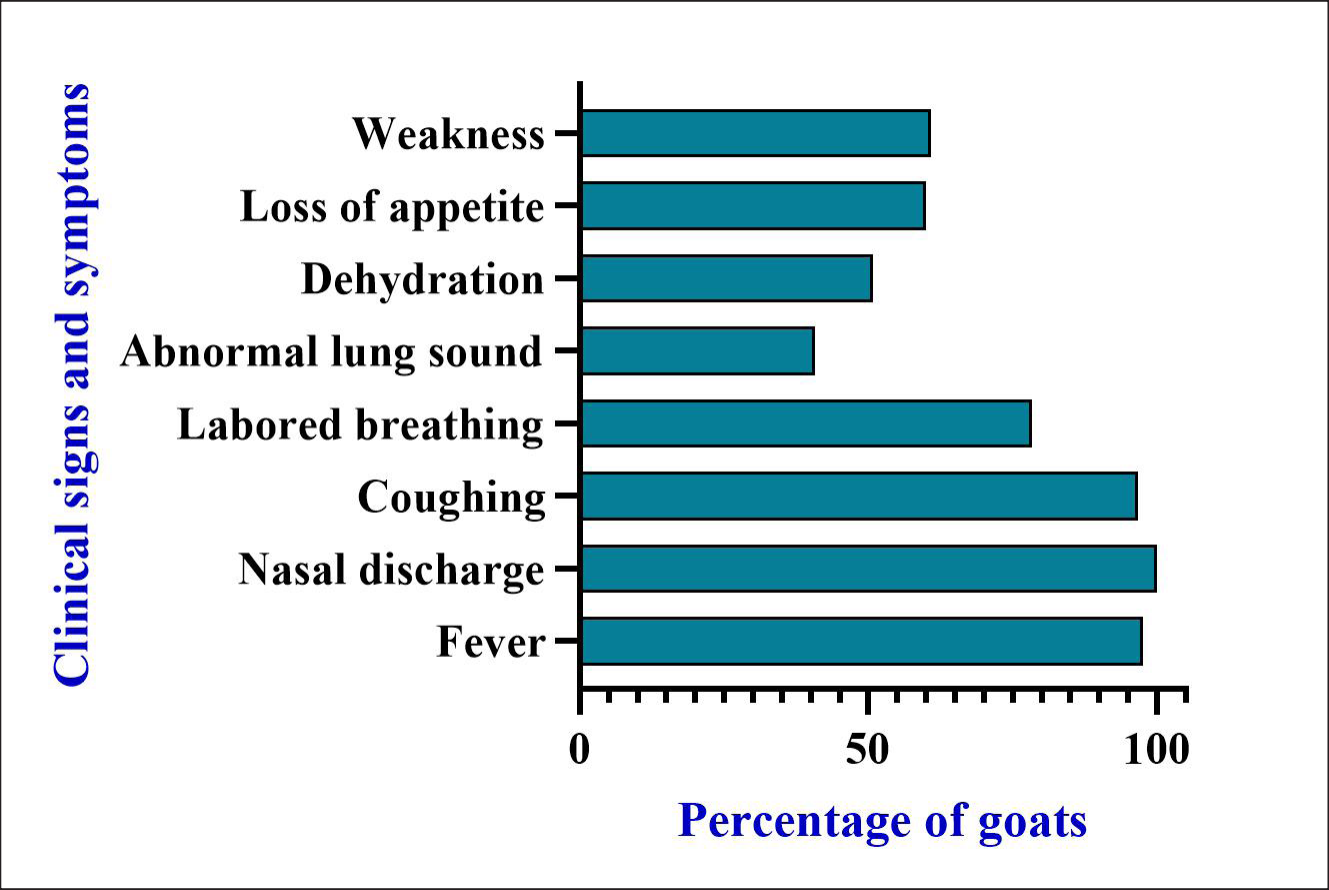
Frequency of diffeent clinical condtions observed in goats during RTI.

Several signs and symptoms were found during the clinical examination of the RTI goats. Among all (*N = *120) RTI cases, most of the goats had a fever (97.5%), nasal discharge (100%), coughing (96.6%), and labored breathing (78.3%). Besides, abnormal sounds (40.8%), like crackles, rales, wheezes, and friction rubs, were detected during auscultation; dehydration (50.8%), weakness (60.8%), and loss of appetite (60%) were present among the affected goats (Fig. 2).

Based on the isolation frequency of *S. aureus* and *E. coli *from RTI goats, the clinical symptoms such as fever (100% and 87.5% goats), dehydration (68.7% and 50% goats), nasal discharge (100% goat in both isolates), and coughing (93.7% and 100% goats) were recorded. Furthermore, most of the *S. aureus- *and *E. coli*-causing RTI-infected goats had labored breathing (93.7% and 87.5%). Moreover,* S. aureus* and *E. coli* infected 50% and 62.5% of goats, respectively, and abnormal sounds such as crackles, rales, wheezes, friction rubs, and grating were detected during auscultation, respectively. Besides, most *S. aureus-* and *E. coli*-infected (68.7% and 62.5%) goats had loss of appetite and weakness, consequently (Table 1).

### AMR pattern of S. aureus

**Figure 3.**
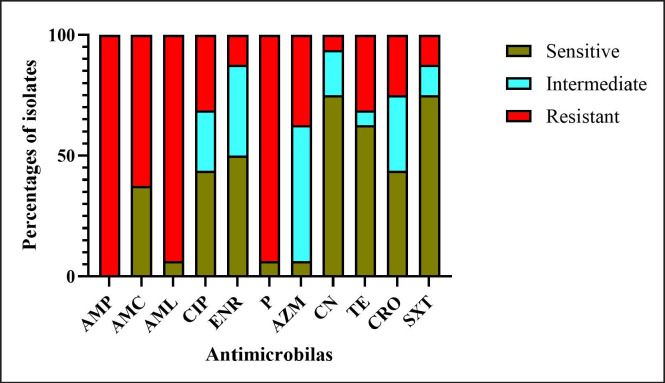
Antimicrobial sensitivity profile of *S. aureus* isolated from RTI goats. Where, AMP: Ampicillin, AMC: Amoxicillin-Clavulanic acid, AML: Amoxicillin, CIP: Ciprofloxaci, ENR: Enrofloxacin, P: Penicillin, AZM: Azithromycin, CN: Gentamycin, TE: Tetracycline, CRO: Ceftriaxone, SXT: Sulfamethoxazole-trimethoprim.

All *S. aureus* positive isolates (*N = *16) were found to be resistant to all classes of antimicrobials. The bacteria exhibited the highest resistance to ampicillin (100%), followed by amoxicillin (93.75%), penicillin (93.75%), amoxicillin-clavulanic acid (62.5%), azithromycin (37.5%), ciprofloxacin (31.25%), tetracycline (31.25%), and ceftriaxone (25%). On the contrary, *S. aureus *was found highly sensitive against sulfamethoxazole-trimethoprim (75%), gentamycin (75%), tetracycline (62.5%), and enrofloxacin (50%) (Fig. 3).

**Table 1.** Sign and symptoms associated with RTI caused by *S. aureus *and *E. coli* in goats found at SAQTVH, Chattogram during the study period.

Sign or symptom	*S. aureus* (*N* = 16)		*E. coli* (*N* = 8)	
	*n*	%	*n*	%
Fever (≥103.6 °F)	16	100	7	87.5
Dehydration (moderate)	11	68.7	4	50
Nasal discharge	16	100	8	100
Coughing	15	93.7	8	100
Respiration (Labored breathing)	15	93.7	7	87.5
Abnormal sound in lungs (crackles, rales, wheezes, friction rubs, and grating sound)	8	50	5	62.5
Feeding (loss of appetite)	11	68.7	5	62.5
Weakness	9	56.2	7	87.5

### AMR pattern of E. coli

**Figure 4.**
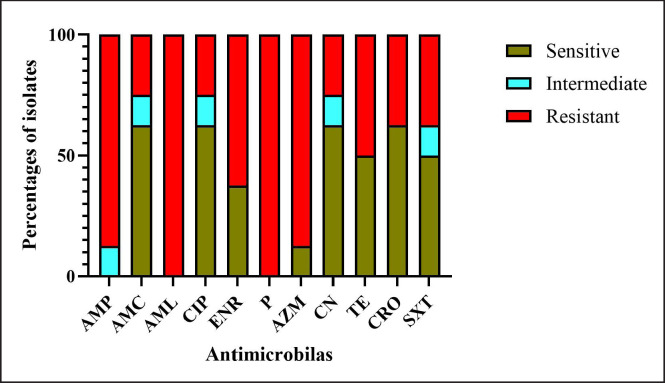
Antimicrobial sensitivity profile of of *E. coli* isolated from RTI goats. Where, AMP: Ampicillin, AMC: Amoxicillin-Clavulanic acid, AML: Amoxicillin, CIP: Ciprofloxaci, ENR: Enrofloxacin, P: Penicillin, AZM: Azithromycin, CN: Gentamycin, TE: Tetracycline, CRO: Ceftriaxone, SXT: Sulfamethoxazole-trimethoprim.

*E. coli* isolates showed the highest resistance rate against both amoxicillin and penicillin (100%), followed by ampicillin (87.5%), azithromycin (87.5%), enrofloxacin (62.5%), tetracycline (50%), ceftriaxone (37.5%), and sulfamethoxazole-trimethoprim (37.5%). On the other hand, 62.5% of *E. coli *isolates were sensitive to amoxicillin-clavulanic acid, ciprofloxacin, gentamicin, and ceftriaxone, and 50% of isolates were sensitive to tetracycline and sulfamethoxazole-trimethoprim (Fig. 4).

### MDR patterns of S. aureus and E. coli

Overall, 43.7% (*N = *7; 95% CI: 2.3–11.6) of *S. aureus* and 62.5% (*N = *5; 95% CI: 1.3–9.4) of *E. coli *isolates were found to be MDR (resistant to at least 3 classes of antimicrobials). Among them, 37.5% (*N = *6) isolates of *S. aureus* and 25% (*N = *2) isolates of *E. coli* were found resistant to 3 classes of antimicrobials (Table 2).

Among six MDR *S. aureus* isolates, two isolates (33.3%) were resistant to beta-lactam, fluoroquinolone, and macrolides, and two isolates (33.3%) were resistant to beta-lactam, fluoroquinolone, and tetracycline. On the other hand, one isolate (16.6%) was resistant to beta-lactam, macrolides, and tetracycline, and one isolate (16.6%) was resistant to beta-lactam, tetracycline, and sulfonamides. Likewise, two *E. coli *isolates were resistant to 3 classes of antibiotics, including beta-lactam, fluoroquinolone, and macrolides. Furthermore, 14.29% (*N = *1) of *S. aureus* and 20% (*N = *1) of *E. coli* exhibited resistance to five antibiotic groups, namely beta-lactam, fluoroquinolone, macrolides, tetracycline, and sulfonamides. In addition, 40% (*N = *2) of *E. coli *isolates displayed resistance against all six groups of antibiotics, namely beta-lactam, fluoroquinolone, macrolides, aminoglycosides, tetracycline, and sulfonamides (Tables 2 and 3).

### Risk factor analysis for RTI caused by S. aureus and E. coli in goats

For *S. aureus*, the rate of infection was higher (10%; *N = *12) in household goats than in farm goats. Adult goats (age > 12 months) were more frequently infected (7.5%; *N = *9) than young goats (age < 12 months). Female goats had a higher infection rate (11.6%; *N = *14) than male goats. Goats with poor body conditions were more prone to infection (11.6%; *N = *14) than those with good ones. Goats reared in a semi-intensive farming system had higher infection rates (8.3%; *N = *10) than those in an intensive system. In addition, unvaccinated goats and those not dewormed were more frequently infected (12.5% and 11.6%; *N = *15 and 14) than vaccinated or dewormed goats (Table 4).

**Table 2.** Overall pattern of multidrug resistance in *S. aureus* and *E. coli* isolated from goats.

Multi-drug resistance antibiotic groups	*S. aureus* *n* (%)	*E. coli* *n* (%)
3	6 (37.5)	2 (25)
4	0 (0)	0 (0)
5	1 (6.25)	1 (12.5)
6	0 (0)	2 (25)

**Table 3.** MDR patterns of *S. aureus* and *E. coli* isolated from goats.

MDR antibiotic groups	Classes of antimicrobials	*S. aureus* *n* (%)	*E. coli* *n* (%)
3	Beta-lactam + Fluoroquinolone + Macrolides	2 (33.3)	2 (100)
	Beta-lactam + Macrolides + Tetracycline	1 (16.6)	0
	Beta-lactam +Tetracycline + Sulfonamides	1 (16.6)	0
	Beta-lactam + Fluoroquinolone + Tetracycline	2 (33.3)	0
5	Beta-lactam + Fluoroquinolone + Macrolides +Tetracycline + Sulfonamides	1 (100)	1 (100)
6	Beta-lactam + Fluoroquinolone + Macrolides + Aminoglycoside + Tetracycline + Sulfonamides	0	2 (100)

In the logistic regression model, female goats were 4.2 times more likely to have tested positive for respiratory infection caused by *S. aureus* than males (95% CI: 0.8-20.8) (*p *= 0.074). Furthermore, the bacterial infection rate was 3.8 times higher in goats with poor BCS compared to those with good BCS (95% CI: 0.7–19.3) (*p *= 0.100). Moreover, the odds ratio for bacterial infection was 4.8 (95% CI: 1–23.6) (*p *= 0.051) in goats that were not dewormed compared to those that received anthelmintic prophylaxis. Finally, goats reared in the semi-intensive system were 2.7 times more prone to *S. aureus* infection than those reared in the intensive housing system (95% CI: 0.8-8.7) (*p *= 0.092) (Table 5).

For *E. coli*, the infection rates in household and farm goats were 5% (*N = *6) and 1.6% (*N = *2), respectively. Local breeds had a higher infection rate of 5% (*N = *6) than Jamnapari goats. Goats in poor health were more susceptible (5.8%; *N = *7) to infection compared to those in good health. Intensively reared goats had a higher infection rate (4.1%; *N = *5) than those reared semi-intensively. Finally, unvaccinated and non-dewormed goats were more frequently infected (5.8% and 4.1%; *N = *7 and 5) than those who were vaccinated against peste des petits ruminants (PPRs) and dewormed (Table 6).

## Discussion

Investigation of *S. aureus* and *E. coli* from caprine RTIs highlights the significant role these pathogens play in goat pneumonia.* S. aureus* is frequently found in the nasal cavity in goats and is considered one of the most common causes of RTI and secondary infection [18,19,25]. In the present study, the occurrence of *S. aureus* was 13.3% in RTI-affected goats. A similar percentage was reported in various geographical locations worldwide; for example, in Bangladesh, the prevalence of *S. aureus* for respiratory infections was around 14%, and in Ethiopia, the prevalence was 7.8% [51,9,49]. However, the carriage rate was comparatively lower than other study findings in Bangladesh conducted by Rashid et al. [44] and Momin et al. [32]; they reported the bacterial prevalence of RTI in goats was 40% and 26%, respectively. Variations in the prevalence of RTI-causing bacteria in small ruminants could be due to host factors, geographical location, animal stress, management practices, transportation, immune status, seasonal variations, and differences in the isolation method [37,11]. In addition, viral infections such as PPR and mycoplasmal infections like *Mycoplasma mycoides* subsp. Capri and *Mycoplasma pneumoniae* can predispose goats to opportunistic infections by *S. aureus* and *E. coli* in cases of caprine RTI [11,19].

**Table 4.** Univariate association between factors and the binary response of *S. aureus *prevalence in goats in Chattogram (*N = *120).

Factor	Category	*S. aureus* *n* (%)	95% CI	*p*-value
Source	Farm (24)	4 (16.6)	4.7–37.3	0.737
	Household (96)	12 (12.5)	6.6–20.8	
Breed	Local (71)	8 (11.2)	4.9–21	0.429
	Jamnapari (49)	8 (16.3)	7.3–29.6	
Age	1-12 months (53)	7 (13.2)	5.4–25.3	0.462
	>12 months (67)	9 (13.4)	6.3–23.9	
Sex	Male (39)	2 (5.1)	0.6–17.3	0.087
	Female (81)	14 (17.2)	9.7–27.2	
BCS	Good (35)	2 (5.7)	0.7–19.1	0.146
	Poor (85)	14 (16.4)	9.3–26.1	
Rearing system	Intensive (65)	6 (9.2)	3.4–19	0.183
	Semi-intensive (55)	10 (18.1)	9.1–30.9	
PPR vaccination	Yes (16)	1 (6.2)	0.16–30.2	0.693
	No (104)	15 (14.4)	8.3–22.6	
Deworming	Yes (38)	2 (5.2)	0.6–17.7	0.090
	No (82)	14 (17.0)	9.6–26.9	

CI = Confidence interval, significance (*p* ≤ 0.05).

**Table 5.** Outputs of multivariate logistic regression model (*S. aureus*) (*N = *120).

Factor	Category	OR	95% CI	*p*-value
Sex	Ref			
	Female	4.2	0.8–20.8	0.074
BCS	Ref			
	Poor	3.8	0.7–19.3	0.100
Deworming	Ref			
	No	4.8	1.0–23.6	0.051
Rearing system	Ref			
	Semi-intensive	2.7	0.8–8.7	0.092

Ref.: Reference value

In our study, the isolation rate of *E. coli* was 6.67% in RTI goats. Similarly, the *E. coli* prevalence in goats RTI was 7.69% and 15% in Egypt and Iraq, as reported by Dutta et al. [18] and Ahmed and Abdullah [3]. However, the previous study reported the prevalence of *E. coli *in the upper respiratory tract of Black Bengal goats was 23% and 25% in Bangladesh, which was much higher than our finding [9,44]. Since *E. coli* acts as an opportunistic pathogen in RTI, which could complicate the disease condition by overlapping infection with other respiratory pathogens such as PPR, *M. pneumoniae*, *Mannheimia* spp*.,* *Pasteurella* spp*.*, and so on [11,47]. Moreover, overcrowding, browsing habits, poor management practices, and immunosuppression might be associated with upper respiratory tract contamination of *E. coli *in goats.

In the study, several clinical conditions, such as fever, nasal discharge, coughing, and labored breathing, were commonly observed in RTI goats. Paul et al. [39] and Momin et al. [32] also revealed closely similar signs and symptoms in goats affected by* S. aureus* and *E. coli* in RTI. This clinical condition and pathogenesis of RTI could be initiated by the multiple virulence factors of bacterial pathogens or might be triggered by pathogen-mediated host-body immune responses.

**Table 6.** Univariate association between factors and the binary response of *E. coli *prevalence in goats in Chattogram (*N = *120).

Factor	Category	*E. coli* *n* (%)	95% CI	*p*-value
Source	Farm (24)	2 (8.3)	1.0–26.9	0.660
	Household (96)	6 (6.2)	2.3–13.1	
Breed	Local (71)	6 (8.4)	3.1–17.4	0.470
	Jamnapari (49)	2 (4.1)	0.4–13.9	
Age	1–12 months (53)	4 (7.5)	2.0–18.2	0.731
	>12 months (67)	4 (5.9)	1.6–14.5	
Sex	Male (39)	4 (10.2)	2.8–24.2	0.435
	Female (81)	4 (4.9)	1.3–12.1	
BCS	Good (35)	1 (2.8)	0–14.9	0.435
	Poor (85)	7 (8.2)	3.3–16.2	
Rearing system	Intensive (65)	5 (7.6)	2.5–17.0	0.725
	Semi-intensive (55)	3 (5.4)	1.1–15.1	
PPR vaccination	Yes (16)	1 (6.2)	0.1–30.2	1.0
	No (104)	7 (6.7)	2.7–13.3	
Deworming	Yes (38)	3 (7.8)	1.6–21.3	0.707
	No (82)	5 (6.1)	2.0–13.6	

CI = Confidence interval, significance (*p* ≤ 0.05).

Antimicrobial sensitivity testing of *S. aureus* isolates revealed that all isolates were resistant against ampicillin, which is similar to the findings of Al Emon et al. [6], Aziz and Lafta [10], and Al Amin et al. [5]. Furthermore, in this study, *S. aureus* isolates exhibited higher resistance against amoxicillin, penicillin, and amoxicillin-clavulanic acid. These observations closely align with the previous findings of Dutta et al. [18] and Momin et al. [32], who reported that isolates obtained from goats were less sensitive to amoxicillin and penicillin. However, the findings of Akter et al. [4], Asaduzzaman et al. [9], and Paul et al. [39] were less similar to our findings because they revealed *S. aureus *isolates were comparatively more sensitive to ampicillin and amoxicillin. However, β-lactam antimicrobials are considered the first choice of drugs to treat the respiratory infection in small ruminants [18,15,14].

Irrational use and constant selective pressure of the antimicrobials could lead to the evolution of resistant strains [7,43]. Moreover, bacterial pathogens may become resistant by acquiring resistant plasmid or chromosomal genes from closely related pathogens through conjugation or horizontal transfer [20,26]. Besides, spontaneous mutation, alteration of antimicrobial binding sites, and antibiotic efflux mechanisms could be associated with the high percentages of resistance in *S.* aureus [7,43,20].

In the current study, *S. aureus* isolates showed higher sensitivity to sulfamethoxazole-trimethoprim (75%) and enrofloxacin (50%). Several previous studies, Al Emon et al. [6] and Momin et al. [32], also found sulfamethoxazole-trimethoprim and enrofloxacin as sensitive drugs for *S. aureus*. In contrast, Akter et al. [4] and Asaduzzaman et al. [9] reported resistance against sulfamethoxazole-trimethoprim in buffalo and Black Bengal goats, respectively.

In this investigation, all *E. coli* isolates displayed resistance to amoxicillin and penicillin. In addition, higher resistance was observed against ampicillin, enrofloxacin, and tetracycline. Several previous studies also reported the higher resistance of *E. coli* against different groups of antimicrobials [4,6,9,47]. It is alarming that gram-negative bacteria like *E. coli* tremendously develop resistance to β-lactam and tetracycline drugs [24]. β-lactam, fluoroquinolone, and tetracycline drugs are most frequently used for therapeutic purposes in the veterinary practices of Bangladesh [43]. Fluoroquinolone and tetracycline have di- and trivalent cations that form a stable complex, which facilitates them to persist in the environment for longer periods [40]. Animals treated with antibiotics continuously store and periodically shed resistant bacteria and thus may transmit them to other animals [12]. Besides transferring resistance genes, bacterial mobile genetic elements such as plasmids, transposons, and integrons also encode enzymes to modify and inactivate various antibiotics [23]. In the present study, *E. coli *isolates were highly sensitive to ceftriaxone, amoxicillin-clavulanic acid, ciprofloxacin, and gentamicin. These results align with the previous findings of Akter et al. [4]. Al Emon et al. [6] also described that *E. coli *isolates were highly sensitive to ciprofloxacin and gentamicin. Conversely, Islam et al. [24] noted higher percentages of *E. coli* were resistant to amoxicillin-clavulanic acid and gentamicin. However, AMR patterns could be changed according to time, geographical location, clinical burden of infectious disease, antimicrobial prescribing practices, and stability and exposure to antimicrobial residues in the animal feed and environment [13].

In this study, 43.7% of *S. aureus* and 62.5% of *E. coli *isolates were found to be MDR that exhibit resistance against a minimum of three groups of antimicrobials. 37.5% of *S. aureus* and 25% of *E. coli* isolates were resistant to three groups of antimicrobials. The resistant groups include beta-lactam, fluoroquinolone, tetracycline, sulfonamides, aminoglycosides, and macrolides. The resistant groups are similar to the previously detected resistant groups reported by Rana et al. [43], Fazal et al. [21], Singh et al. [47], and Das et al. [17]. MDR is considered a significant challenge for the clinical management of infectious diseases, particularly for food animals [42,21]. It may arise due to indiscriminate use and not maintaining the withdrawal period of antibiotics [2]. In veterinary hospitals of Bangladesh, there is inadequate disease diagnostic capacity, workforce, and logistics. Therefore, antibiotics are prescribed based on history and clinical signs [15]. Moreover, the emergence of MDR also poses a significant threat to public health, as residues of these antibiotics in meat and milk from slaughtered food animals could cause the emergence of drug-resistant bacteria in consumers [50].

Therefore, regular monitoring for the emergence of AMR is crucial for selecting appropriate and effective antimicrobials for therapeutic applications and controlling the further spread of resistant pathogens. The identification of MDR strains underscores the growing challenge of AMR in goats, which complicates treatment protocols and compromises animal health. Furthermore, controlled antibiotic use through strict regulation, educating farmers and veterinary professionals through training, awareness campaigns, judicious use of antimicrobials, and increasing logistics to veterinary hospitals for improving diagnostic capacity are essential to mitigate MDR.

In the present study, we identified multiple factors associated with the carriage of pathogens in RTI in goats. The incidence of *S. aureus* infection was observed to be higher (2.7 times) in the goats that were reared in a semi-intensive farming system compared to an intensive farming system. When goats graze outside or contact diseased animals, they may get exposed to infectious pathogens, sudden climate change such as heat and rain, and stress conditions could encourage the opportunistic pathogen to establish primary infection in the respiratory tract [27,31]. RTI caused by *S. aureus* was significantly higher (OR = 4.2) in female than male goats. These findings are supported by several national studies [9,33, 39]. Nath et al. [38] reported that female goats are more susceptible to infectious diseases than males. The higher prevalence of RTI in female goats might be due to higher disease susceptibility, various stressors (pregnancy, lactation, and environmental), and a higher female population in herds [35]. Moreover, respiratory infection caused by *S. aureus* was 3.8 times higher in goats with poor body condition (thin). The finding is supported by Sah et al. [46] and Islam et al. [25], who reported that poor BCS sheep and goats were more susceptible to infectious diseases like pneumonia. An opposite finding was reported by Paul et al. [39]. They showed that *S. aureus* prevalence was higher in goats with good BCS than in poor BCS. The association between loss of BCS and the occurrence of diseases is complex [30]. However, poor body condition weakens the immune system and reduces the capability to defend against infectious pathogens [34]. *Staphylococcus aureus* commonly resides in the upper respiratory mucosa and can cause disease in individuals with weakened immune systems [11]. Our study also revealed that RTI was 4.8 times higher in goats with no deworming record. Deworming status and health condition are correlated to each other. Although endoparasitic and ectoparasitic diseases are less responsible for mortality, they significantly impact a goat’s growth and reproductive capability [8]. Parasitic infection increases the susceptibility to bacterial and viral diseases [22]. In tropical and subtropical countries, goats often become dehydrated. Parasitic infection may lead to fluid imbalance and accumulation in the lung tissue spaces, which hinders the pulmonary defense mechanism. Consequently, other pathogenic microorganisms establish infection [1,29].

There were some limitations in the current study. Although several bacterial and viral pathogens are also responsible for respiratory infection in goats, we investigated only the two most common opportunistic pathogens due to our resource constraints. We were unable to perform any molecular tests to characterize *S. aureus* and *E. coli* isolated from respiratory infections due to budget constraints. Moreover, in the logistic regression model, the *p*-values for certain variables were slightly higher than 0.05. This could be due to a small sample size, potentially reducing the statistical power of the test. A detailed epidemiological investigation will be performed in the future study to elucidate the other related factors that can facilitate the design of effective control strategies against respiratory pathogens.

## Conclusion

In the present study, the occurrence of *S. aureus* and *E. coli* in RTI goats was 13.3% and 6.67%, respectively. High fever with nasal discharge, coughing, and labored breathing are the most frequent clinical conditions recorded in the RTI goats. A high proportion of *S. aureus* and *E. coli* isolates were resistant to commonly used antibiotics such as penicillin, ampicillin, amoxicillin-clavulanic acid, and azithromycin. Moreover, a significant percentage of isolates were found in MDR. Rearing system, poor body condition, sex, and deworming status were identified as important determinants for the carriage of *S. aureus* in respiratory diseases in affected goats. By identifying high resistance rates and MDR pathogens to commonly used antimicrobials, the study highlights the urgent need for more prudent antimicrobial use in veterinary clinical practices. Therefore, a clear call to action for policy development and comprehensive research regarding respiratory pathogens and exploring their resistance patterns with associated factors is of utmost importance to enhance the clinical management and control of RTI goats.

## List of abbreviations

AMC, amoxicillin-clavulanic acid; AML, amoxicillin; AMP, ampicillin; AMR, antimicrobial resistance; AZM, azithromycin; BCS, body condition score; CI, confidence interval; CIP, ciprofloxacin; CLSI, clinical and laboratory standard institute; CN, gentamycin; CRO, ceftriaxone; CVASU, chattogram veterinary and animal sciences university; *E. coli*, *Escherichia coli*; EMB, eosin methylene blue; ENR, enrofloxacin; I, intermediate; MDR, multidrug-resistant; MSA, mannitol salt agar; MR, methyl red; P, penicillin-G; R: resistant; ROC, receiver operating characteristic; RTI, respiratory tract infection; S, sensitive; *S. aureus*, *Staphylococcus aureus*; SAQTVH, sa quaderi teaching veterinary hospital; SXT, sulfamethoxazole-trimethoprim; VP, voges proskauer.
